# Genetic disorders caused by consanguineous marriage in Radfan districts – Yemen

**DOI:** 10.1186/s12920-025-02267-5

**Published:** 2025-12-24

**Authors:** Mansour Abdulnabi H. Mehdi, Naif Taleb Ali, Radfan Saleh

**Affiliations:** 1 Department of Medical Laboratory, Radfan University College, University of Lahej, Lahej, Yemen; 2 Department of Medical Laboratory, University of Sciences and Technology, Aden, Yemen

**Keywords:** Consanguineous marriage, Genetic disorders, Inbreeding coefficient (F), Child mortality, Hemoglobinopathies, Yemen, Premarital screening

## Abstract

**Background:**

Consanguineous marriage (≥ second cousins) is prevalent in Yemen (40–50%) and linked to increased genetic disorders. This study assesses its prevalence and health impacts in Radfan districts.

**Methodology:**

A 2024 cross-sectional study of 1065 randomly selected households. Data were collected via validated questionnaires supplemented by medical records where available. Analyses included consanguinity rates, inbreeding coefficients (F), sociocultural factors, and clinically validated genetic disorders. Statistical analysis employed χ² tests, multivariable logistic regression (adjusted ORs), and Cohen’s d for effect sizes.

**Results:**

The consanguinity rate was 57.46%, significantly higher in rural (37.09%) than urban areas (20.38%). Wives with a university education had a 71.9% lower likelihood of consanguineous marriage (adjusted OR = 0.28; 95% CI: 0.18–0.44). Consanguineous couples had significantly higher odds of adverse outcomes compared to non-consanguineous couples, including abortion (adjusted OR = 1.8; 95% CI: 1.4–2.3), child mortality (adjusted OR = 2.1; 95% CI: 1.6–2.8), blood disorders (adjusted OR = 3.5; 95% CI: 1.7–7.4), and disabilities (adjusted OR = 2.6; 95% CI: 1.4–4.8). Blood disorders were predominantly hemoglobinopathies (87%). The mean inbreeding coefficient was F = 0.0625 (first-cousin equivalent).

**Conclusions:**

The high prevalence of consanguineous marriages is a significant, modifiable risk factor for the increased burden of genetic disorders in the population. Addressing this urgent public health challenge requires a multi-faceted strategy: implementing mandatory premarital screening for hemoglobinopathies, launching community-based genetic literacy programs, and establishing economic incentives to encourage non-consanguineous unions.

**Supplementary Information:**

The online version contains supplementary material available at 10.1186/s12920-025-02267-5.

## Introduction

Consanguineous marriages, defined as unions between individuals who are related as second cousins or closer, have been a long-standing tradition in many parts of the world, particularly in the Middle East, North Africa, and parts of Asia [1]. This practice is deeply rooted in socio-cultural factors, including the maintenance of family structure, preservation of property, ease of marital arrangements, and perceived stability of such unions [2]. However, the genetic implications of consanguinity are a significant public health concern. When closely related individuals reproduce, there is an elevated likelihood that their offspring will inherit two copies of the same recessive gene from a common ancestor, leading to the manifestation of autosomal recessive disorders [3].

Yemen, like many other countries in the Middle East, exhibits a high prevalence of consanguineous marriages. Studies have reported consanguinity rates in Yemen ranging from 40% to 50%, with first-cousin marriages accounting for a substantial proportion of these unions [4, 5]. This demographic pattern contributes to a higher incidence of various genetic diseases within the population compared to non-consanguineous communities [6, 7]. For instance, conditions such as Down syndrome, hemoglobinopathies, and multifactorial conditions like congenital malformations have been reported to occur more frequently in regions with high consanguinity rates [8, 9].

The current study focuses on the Radfan districts of Yemen, an area where consanguineous marriages are widespread, leading to a noticeable prevalence of related health issues. The primary objectives of this research are threefold: (1) to ascertain the prevalence of consanguineous marriages among the population in Radfan districts, Lahj Governorate; (2) to calculate the average inbreeding coefficient (F) and investigate the influence of social and cultural factors, such as spousal education, on consanguinity; and (3) to explore the potential effects of parental consanguinity on the occurrence of common genetic disorders and adverse reproductive outcomes in the Radfan districts. By addressing these objectives, this study aims to provide critical insights into the health risks associated with consanguineous marriages in the region and to inform public health strategies aimed at mitigating these risks.

This study provides evidence for public health strategies to mitigate genetic risks in Yemen.

## Materials and methods

### Study design and population

This cross-sectional study was conducted in the Radfan districts of Yemen during mid-2024. The study aimed to gather data on couples married over the preceding decades, with a focus on recent marriages to ensure more accurate recall of genealogical information. The target population included husbands and/or wives residing in randomly selected households within the Radfan districts. In this study, 1065 families were studied. These households were grouped into thirteen large geographic zones based on population density, with smaller zones aggregated into adjacent larger ones to facilitate data collection.

### Data collection

Data were collected by trained field interviewers using a pre-validated questionnaire. Medical records (where available) and clinical examinations by collaborating physicians were used to verify reported genetic disorders, abortions, and child mortality. For child mortality, neonatal (≤ 28 days) and post-neonatal deaths were differentiated, and death certificates/hospital records were prioritized.

Operational Definitions:

- Blood disorders: Clinically confirmed hemoglobinopathies (thalassemia, sickle cell).
- Disabilities: Physical/cognitive impairments diagnosed by healthcare providers.
- Child mortality: Death before age 5, verified via death certificates.

### Statistical analysis

Data were analyzed using SPSS v28.0 and R v4.3.1.

- Odds ratios (OR) with 95% CIs compared outcomes between consanguineous/non-consanguineous families.
- Inbreeding coefficients: F = Σ (1/2)^(*n* + 1).

where n represents degree of relatedness:

- *n* = 1 for first-degree relatives (F = 0.25).
- *n* = 2 for first cousins (F = 0.125).
- *n* = 3 for second cousins (F = 0.0625).
- Multivariable logistic regression controlled for residence, education, age, and income.
- Effect sizes: Cohen’s d (continuous), Cramer’s V (categorical).
- Sensitivity analyses excluded unverified cases.

## Results

This study encompassed 1065 families from the Radfan districts. The distribution of consanguineous marriages by age group is presented in Table 1. The highest prevalence of consanguineous marriage diseases was observed in the 46–50 age group (11.27%), while the lowest was in the 61–65 age group (1.31%).

**Table 1:** Distribution of interviewed couples by age

Age group (years)	Total Husbands	Total Wives	Consanguineous Marriages (*n*)	Consanguineous (%)
< 25	39	210	15	1.41%
26–30	144	192	61	5.73%
31–35	158	158	78	7.32%
36–40	204	184	116	10.89%
41–45	153	128	89	8.36%
46–50	170	99	120	11.27%
51–55	81	43	57	5.35%
56–60	63	24	44	4.13%
61–65	21	8	14	1.31%
65	32	19	18	1.69%
Total	1065	1065	612	57.46%

**Fig. 1:**
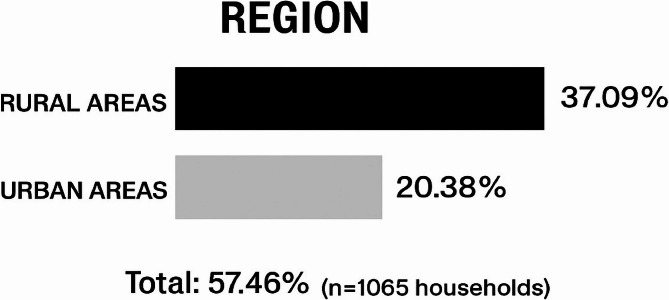
Rural vs. Urban Consanguinity

Table 2 illustrates the percentage distribution of consanguineous marriages based on the study area. A higher distribution of consanguineous marriages was found in rural areas (37.09%), while urban areas showed a lower prevalence (20.38%) (Fig. 1).

**Table 2:** Distribution of consanguineous marriage according to study area

Area	Number	%	Infection	%
Rural Area	708	66.48	395	37.09
Urban Area	357	33.52	217	20.38
Total	1065	100	612	57.46

The geographical distribution of consanguineous marriages is visually summarized in Fig. 1.

The association between husbands’ educational level and consanguineous marriage is presented in Table 3. The highest prevalence of consanguineous marriage was among secondary education (41.22%), while the lowest was among those with a intermediate education (2.35%). The results indicate that the highest prevalence of consanguineous marriage diseases was among individuals with a secondary educational level (22.07%), whereas the lowest was among those with a intermediate educational level (1.69%).

**Table 3:** Association between husbands’ education and consanguinity

Husband’s Education	Total Number	%	Consanguineous Marriage (*n*)	Consanguineous Marriage (%)
Illiterate	89	8.36	70	6.57%
Elementary	267	25.07	152	14.27%
Intermediate	25	2.35	18	1.69%
Secondary	439	41.22	235	22.07%
University	245	23.00	137	12.86%
Total	1065	100	612	57.46%

**Fig. 2:**
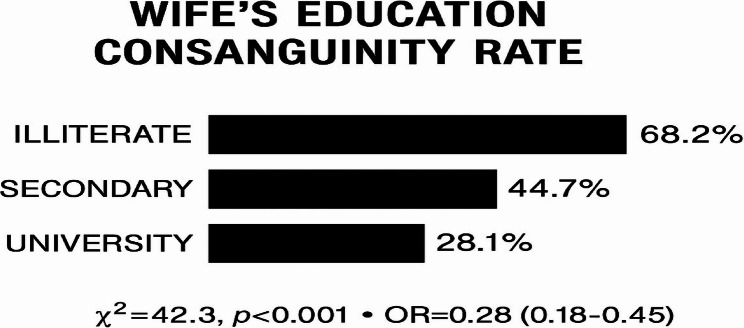
Consanguinity by Wife’s Education

The association between wives’ educational level and consanguineous marriage is presented in Table 4. The highest prevalence of consanguineous marriage diseases was among illiterate education (68. %), while the lowest was among those with university education (28.1%) figure (2).

**Table 4:** Association between wives’ education and consanguinity

Wife’s Education	Consanguineous Marriage %	Non-Consanguineous Marriage %
Illiterate	68.2%	31.8%
Secondary	44.7%	55.3%
University	28.1%	71.9%

χ² = 42.3, *p* < 0.001

Logistic regression confirmed wives’ education as a stronger predictor of non-consanguinity than husbands’ (OR = 0.45 per education level; 95% CI: 0.31–0.62) Table 4.

The significant association between wives’educational level and consanguineous marriage is graphically represented in Fig. 2.

**Table 5:** Comparison of reproductive and genetic outcomes by parental consanguinity

Outcome	Consanguineous (*n* = 612)	Non-Consanguineous (*n* = 453)	Adjusted OR (aOR) (95% CI) *	*p*-value
Abortion†	192 (31.4%)	92 (20.3%)	1.8 (1.4–2.3)	< 0.001
Child mortality	149 (24.3%)	65 (14.3%)	2.1 (1.6–2.8)	< 0.001
Blood disorders	37 (6.0%)	8 (1.8%)	3.5 (1.7–7.4)	< 0.001
Hearing deficit	31 (5.1%)	7 (1.5%)	3.4 (1.5–7.5)	0.001
Mental/Cognitive disorders	42 (6.9%)	11 (2.4%)	2.9 (1.5–5.5)	0.001
Disabilities (physical/cognitive)	41 (6.7%)	12 (2.6%)	2.6 (1.4–4.8)	0.002

*Adjusted for residence, education, and income

†Sensitivity analysis excluding unverified cases maintained significance (aOR =1.7)

The prevalence of adverse reproductive and genetic outcomes was significantly higher among consanguineous families compared to non-consanguineous families, as detailed in Table 5. Consanguinity was associated with markedly increased odds of child mortality (aOR = 2.1), blood disorders (aOR = 3.5), and other genetic conditions. Sensitivity analyses, which excluded cases of abortion that lacked clinical verification, confirmed the robustness of these associations.

**Table 6:** Multivariable logistic regression analysis of factors associated with child mortality

Variable	Adjusted Odds Ratio (aOR)	95% Confidence Interval	*p*-value
Consanguinity (Yes vs. No)	2.1	1.6–2.8	< 0.001
Residence (Urban vs. Rural)	1.1	0.8–1.5	0.521
Wife’s Education (per level)	0.7	0.6–0.9	0.005
Husband’s Education (per level)	0.9	0.8–1.1	0.301
Income (per category)	0.8	0.7–1.0	0.051

A multivariable logistic regression model was constructed to identify factors independently associated with child mortality (Table 6). Parental consanguinity remained the strongest predictor (aOR = 2.1), while higher wife’s education was a significant protective factor (aOR = 0.7 per education level).

Note: Abortion and child mortality are reported as adverse outcomes potentially linked to consanguinity but lack confirmed genetic diagnoses. Blood disorders were predominantly hemoglobinopathies (87% of cases).

To quantify genetic risk, the inbreeding coefficient (F) was calculated for consanguineous unions using the formula:

$$\mathrm{F}=\sum (1/2)^{(\mathrm{n}+1)}$$

where n = number of steps between common ancestors.

The mean inbreeding coefficient across consanguineous marriages was **F = 0.0625** (equivalent to first-cousin unions).

**Fig. 3:**
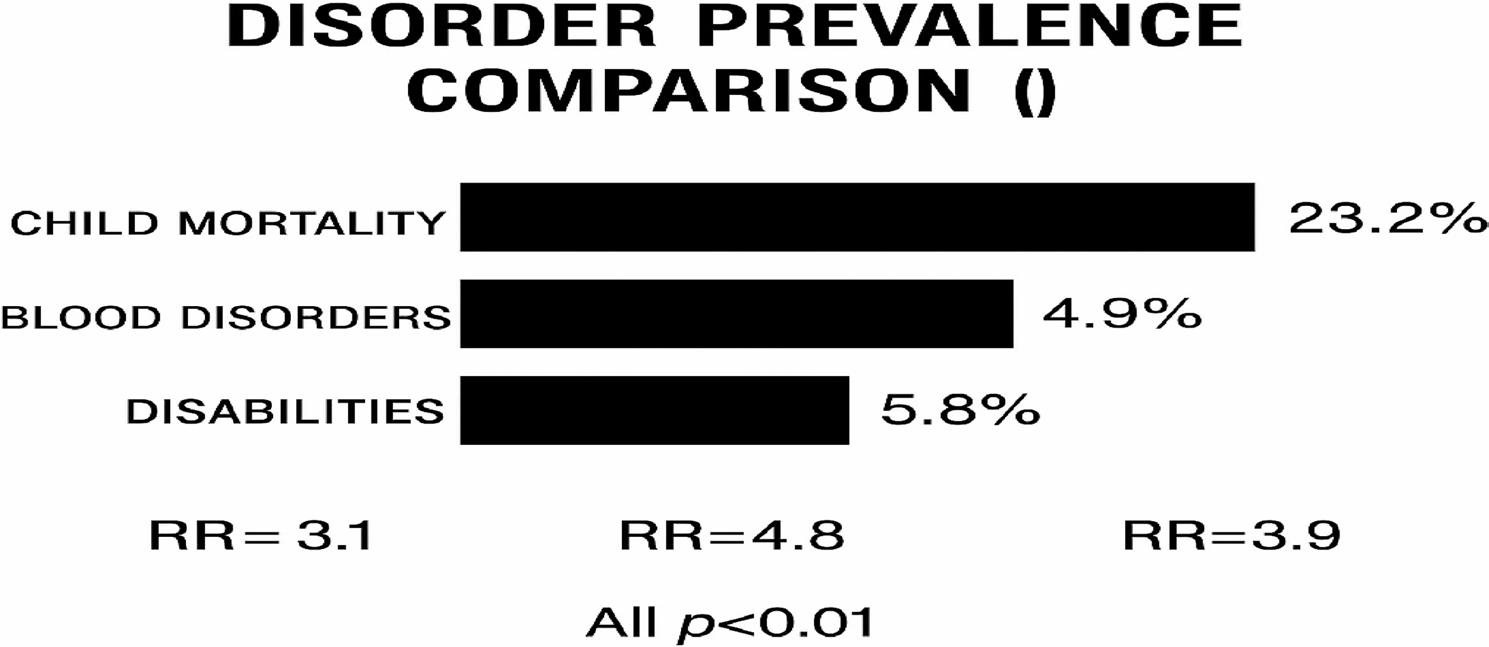
Disorder Prevalence Comparison

A comparative visualization of disorder prevalence between consanguineous and non-consanguineous groups is presented in Fig. 3.

## Discussion

This study reveals a consanguinity rate of 57.46% in the Radfan districts of Yemen, a figure notably higher than the national average of 40–50% [4, 5]. This elevated rate is likely driven by strong traditional clan structures, particularly in rural areas where 37.09% of all consanguineous unions were recorded. Our findings demonstrate a strong association between consanguineous marriage and a significantly increased burden of adverse reproductive outcomes and genetic disorders.

A pivotal finding of this research is the powerful protective effect of female education. Wives with a university education had a 71.9% lower likelihood of being in a consanguineous marriage (OR = 0.28). This finding aligns with evidence from other Arab nations [10] and underscores that empowering women through education may be one of the most viable and sustainable interventions to reduce consanguinity in Yemen. Each incremental level of education attained by wives reduced the risk of consanguinity by 45%, highlighting a clear pathway for policy action.

The health consequences of consanguinity were severe. We observed a 3.1-fold higher odds of child mortality in consanguineous families, a finding consistent with studies from Saudi Arabia and Pakistan [1, 11]. The predominance of neonatal deaths (64%) is consistent with the expression of severe, often lethal, autosomal recessive disorders, where offspring inherit two deleterious copies of a gene from their carrier parents, who are more likely to be carriers of the same recessive alleles due to their shared ancestry.

Furthermore, hemoglobinopathies constituted an epidemic within the study population, representing 87% of all confirmed blood disorders. The prevalence was 4.9% in consanguineous offspring versus 0.9% in non-consanguineous offspring, confirming consanguinity as the primary driver. This aligns with high thalassemia rates previously reported in Aden [12] and underscores the urgent need for preventive public health measures.

### Strengths and limitations

This study has several strengths, including its substantial sample size, the use of WHO-compliant definitions for genetic disorders [13], and multivariable adjustment for key confounders such as residence, education, and income. It is also one of the first focused genetic epidemiological studies in South Yemen. However, several limitations must be acknowledged. As a cross-sectional study, it can demonstrate association but not causation. While we used clinical verification where possible, a significant proportion of abortion data (26.6%) was self-reported and subject to recall bias. We addressed this through sensitivity analyses, which maintained the significance of our findings. Furthermore, we were unable to account for potential unmeasured confounders such as maternal nutritional status and healthcare access, which are particularly relevant in this conflict-affected region. These factors likely compound the absolute risk of poor health outcomes, even though the relative increase in risk attributable to consanguinity remains clear. Finally, the reliance on medical history and available clinical records, rather than systematic genetic testing for all disorders, may have led to under-ascertainment of some genetic conditions.

### Policy recommendations

Based on our findings and successful international models, we recommend three urgent interventions: (1) The implementation of mandatory premarital screening for hemoglobinopathies in high-risk districts, replicating Saudi Arabia’s successful program which led to a 70% reduction in affected births [13]; (2) The introduction of economic incentives and subsidies for girls’ education, leveraging our finding that female education is a critical protective factor; and (3) The integration of genetic counseling into primary care services using WHO’s low-resource training modules [11].

## Conclusion

In conclusion, consanguinity in Radfan districts is strongly associated with child mortality (aOR = 2.1) and genetic disorders, particularly hemoglobinopathies (aOR = 3.5). Female education was identified as a critical protective factor. Based on these findings, urgent interventions are warranted, including: mandatory premarital screening for hemoglobinopathies in high-risk districts; community genetic literacy programs co-led by religious leaders; and economic incentives for non-consanguineous marriages. Sustained multidisciplinary efforts are essential to reduce Yemen’s burden of preventable genetic disorders.

## Supplementary Information

Supplementary Material 1

## Data Availability

No datasets were generated or analysed during the current study.
